# The Effect of an Educational Program on the Knowledge and Attitude of Medical Sciences Students About Social Determinants of Health in Iranian University Students: A Quasi‐Experimental Study

**DOI:** 10.1002/hsr2.70182

**Published:** 2024-11-06

**Authors:** Mostafa Amini‐Rarani, Athar Omid, Mehdi Nosratabadi

**Affiliations:** ^1^ Health Management and Economics Research Center Isfahan University of Medical Sciences Isfahan Iran; ^2^ Department of Medical Education, Medical Education Research Center Isfahan University of Medical Sciences Isfahan Iran; ^3^ Social Determinants of Health Research Center Isfahan University of Medical Sciences Isfahan Iran

**Keywords:** attitude, educational intervention, knowledge, medical education, quasi‐experimental studies, student

## Abstract

**Background and Aims:**

Given the importance of social determinants on health outcomes, training medical sciences students in addressing social determinants of health can enhance their effectiveness and social accountability. The aim of this study was to assess the effect of an educational program on the knowledge and attitude of medical sciences students regarding social determinants of health.

**Methods:**

Using a one‐group pretest‐posttest quasi‐experimental design, this study selected 200 students from a medical university in Iran through simple random sampling. A tailored educational intervention, grounded in the conceptual framework for action on social determinants of health, was implemented. To assess the program's effect, data were collected using a validated questionnaire that measured the students' knowledge and attitudes towards social determinants of health both at baseline (pretest) and 1 month following the intervention (posttest).

**Results:**

The application of the Wilcoxon Signed‐Ranks Test revealed a significant increase in the median scores for knowledge and attitude postintervention. Specifically, the posttest median scores were significantly elevated compared to the pretest knowledge score (*Z* = −11.89, *p* < 0.001) and attitude score (*Z* = −11.60, *p* < 0.001). This indicates that the educational intervention significantly improved the students' knowledge and attitudes concerning social determinants of health.

**Conclusion:**

The study outcomes suggest that educational interventions focused on social determinants of health effectively improve students' knowledge and attitudes. We recommend integrating such programs into the medical sciences curriculum and experiential training. By doing so, we can better prepare future healthcare professionals to address social determinants of health‐related issues. This approach has the potential to reduce health disparities and also addresses broader social challenges affecting population health.

## 1 Introduction

The World Health Organization (WHO) characterizes social determinants of health (SDH) as the conditions in which individuals are born, grow up, work, and reside. This concept encompasses a diverse array of economic, political, and social factors, as well as norms and policies, all of which influence daily living conditions [1].

Health sciences universities are increasingly expected to embrace a prominent social mission as part of their organizational objectives. Educators within these institutions are encouraged to create educational materials that not only respond to societal needs but also delve deeply into addressing health disparities [2]. In contrast, the concept of social accountability in healthcare demands that educational institutions—particularly those in the health sciences—fulfill their responsibility to the community. This responsibility encompasses ensuring that social needs are met through dedicated research, comprehensive education, and the provision of essential services [3]. Pinpointing curriculum areas that emphasize SDH can significantly boost awareness and cultivate a positive mindset among medical students regarding their social accountability [4].

By increasing health sciences students' awareness of SDH, we can foster a more positive attitude toward this crucial issue. This heightened awareness, driven by factors such as perceived significance and importance [5], enables a shift in focus—moving beyond clinical aspects to address patients' non‐clinical needs. Ultimately, this approach promotes a more holistic and patient‐centered approach to healthcare.

Undoubtedly, equipping health sciences students with the ability to recognize broader social contexts—contexts that influence the emergence of diseases and health complications—is paramount. This comprehensive approach proves indispensable for effectively managing and understanding health outcomes [6]. For example, a study highlighted the efficacy of video‐based curriculum materials focused on SDH. These materials significantly improved medical students' understanding of critical issues, including domestic abuse and depression, within the context of a pediatric day clinic [7]. This suggests that the utilization of multimedia educational resources emerges as a potent tool—one that not only engages but also enriches students' comprehension of complex health issues.

Research findings emphasize the significance of integrating SDH principles into health sciences curricula [8, 9]. However, mere inclusion of SDH content falls short of guaranteeing that students will graduate with the capacity to apply these concepts effectively in community health contexts. Therefore, it becomes crucial not only to teach SDH‐related topics but also to transform students' awareness and attitudes toward SDH. This transformation is essential for adequately preparing them to address SDH in their future professional practice [10, 11]. In Iran, health sciences curricula often insufficiently address of SDH [12], primarily due to the prevailing emphasis on a biological and medical approach in health education. Consequently, beyond mere curriculum revisions, it becomes imperative to devise effective methods for teaching SDH concepts to medical students. These methods play a pivotal role in heightening awareness and reshaping students' attitudes toward SDH. Strategies could encompass diverse educational courses and experiential learning opportunities centered around SDH [13, 14]. While numerous studies have explored the implementation of SDH‐focused curricula, there has been less emphasis on evaluating the impact of these educational programs in enhancing students' knowledge and attitudes. Thus, assessing both content and outcomes of such initiatives is crucial to ensure their effective contribution to the education of health sciences students [15, 16].

Research consistently underscores the positive impact of educating students about SDH on their knowledge base [17, 18]. While studies have extensively analyzed student engagement and satisfaction, the extent to which education influences attitudes and behaviors remains less explored [19]. Remarkably, in Iran, this specific aspect of SDH education has yet to be thoroughly investigated. This research gap presents a valuable opportunity for future studies to assess how SDH education shapes the attitudes and behaviors of health sciences students within the country. By designing targeted educational interventions, with a focus on enhancing medical students' awareness and fostering a positive attitude toward SDH, we lay the groundwork for applying this knowledge to improve patient health and, ultimately, societal well‐being. Consequently, the primary aim of our study was to assess the effectiveness of an educational program in enhancing medical science students' knowledge and attitudes regarding SDH.

## 2 Methods

### 2.1 Study Design and Setting

This study employed a one‐group pretest‐posttest quasi‐experimental design to assess the effect of an educational program. This research was carried out at the Isfahan University of Medical Sciences, which is located in the center of Iran. There are 10 schools at this university. A variety of academic levels, degrees, and training programs are available at these schools, including Bachelor of Science (BSc), Master of Science (MSc), Doctor of Philosophy (PhD), Post‐Doc, Doctor of Medicine (MD), Doctor of Dental Surgery (DDS), Pharmacy, fellowship, subspecialty, and specialty. Furthermore, the university operates health and treatment networks in the respective Province, according to the Iranian health system [20]. The Health and Treatment Network is the highest level of service delivery for public health and treatment. This network coordinates the activities of the Province's urban and rural health centers.

### 2.2 Research Sample

Students from the BSc, MSc, PhD, and MD degree levels took part in the study. Inclusion criteria for student participation was determined based on the following criteria: (1) enrollment in the fifth semester for BSc and MD students, and the third semester for MSc and PhD students; (2) attendance in the classroom setting; and (3) absence of any academic or medical leave. Exclusion from the study applied to students who did not give informed consent, failed to fill out both the pre‐test and posttest questionnaires, or did not participate in all the sessions of the intervention

Using a simple random sampling technique, we selected a sample of 200 students from a total population of 1582. This selection was based on the established formula for determining sample size in studies involving paired continuous data [21].

$$N_{\mathrm{Pairs}}=\frac{2{\left(z_{1-\alpha /2}+z_{1-\beta }\right)}^{2}}{{{∆}^{2}}_{\mathrm{Continous}}}+\frac{z_{1-\alpha /2}^{2}}{2}.$$

Using a significance level of 5%, the power of test of 80%, and the standardized effect size of 0.3, the sample size was estimated as follows:

$$n=\frac{2{\left(1.96+0.842\right)}^{2}}{0.3^{2}}+\frac{1.96^{2}}{2}≅200.$$

To ensure a representative sample, students was selected from the entire student body, we began by obtaining official lists of BSc and MD students in their fifth semester, as well as MSc and PhD students in their third semester, from the vice‐chancellery of education. These lists were then transferred into Excel. Subsequently, the RANDBETWEEN function in Excel was employed to randomly select the sample students. In the recruitment phase, these students were contacted via phone and invited to partake in the study, with the incentive of a mobile internet gift card offered as motivation

### 2.3 Research Procedure

The following four steps were taken:

1. *Developing intervention protocol*
  An educational intervention protocol was developed in accordance with a well‐known conceptual framework for action on the SDH [22] that was endorsed by WHO commission on SDH. This education intervention includes a variety of topics related to SDH concepts, history, definitions, and models.
  SDH are defined as the conditions within the social, physical, and economic environments where individuals are born, grow, live, work, and age. These determinants encompass a wide range of factors, including but not limited to healthcare accessibility. They are shaped by the policies, programs and institutions and other aspects of the social structure including the government and private sectors.
  The history of social perspectives on health was explained by tracing back to several milestones: the 1978 Alma‐Ata Declaration that ignited the Health for All campaign, the establishment of the primary healthcare model, the 1986 Ottawa Charter that advanced health promotion, the recognition in the late 1990s and early 2000s of the failure of existing health policies to reduce inequities, the shift towards strategies focused on equity, and the report of the WHO Commission on SDH in 2008.
  The intervention protocol involves various models of social determinants of health. These included the Dahlgren‐Whitehead rainbow model, which was introduced in 1991 to illustrate the layers of influence on health; Diderichsen's framework, which in 1998 delineated the mechanisms driving health disparities; Mackenbach's model, which explored the interplay of selection and causation in health outcomes; and the Brunner, Marmot, and Wilkinson model, which examined the myriad factors affecting health throughout life course and the Bullseye model of social determinants of health. Also, in alignment with the WHO Commission on Social Determinants of Health, the intervention materials addressed: (1) structural determinants, which encompass the socioeconomic and political contexts, including governance, macroeconomic policies, public policies, societal culture, and values, as well as individual socioeconomic positions marked by education, occupation, income, social class, ethnicity/race, and gender; (2) intermediary determinants, which cover environmental conditions, socio‐psychological factors, behavioral patterns, biological elements, and the health system itself.
  In addition, a 37‐min video clip about SDH made by Iran's Ministry of Health and Medical Education was shown. The learning objectives were to provide information on SDH concepts and their determinants. The video is structured into three primary sections: (1) an exploration of the definitions and significance of social determinants in health; (2) a discussion on the indicators of social determinants and their impact on individual health outcomes; and (3) an examination of the responsibilities and influences of governmental bodies, policymakers, and medical academic institutions concerning social determinants of health.
2. *Pretest*
  The pretest began in November of 2019. In this step, students were invited to complete a previously validated questionnaire about medical science students' knowledge and attitudes toward SDH [23]. to obtain baseline data before educational intervention. In fact, the questionnaire was administered before the educational intervention in this step. The questionnaire is divided into three sections: demographic, knowledge, and attitude. Section I contains demographic data, gender, marital status, academic level, and way of life. Section II has 18 multiple‐choice questions that assess students' knowledge of various aspects of SDH. Section III consists of 15 questions on a 4‐point Likert scale to assess the student's attitude toward SDH. In the case of knowledge and attitude questions, we asked students to select the correct answer and rate it on a scale of 1 (*disagree*) to 4 (*agree*). It was also made clear to the students that their participation was entirely voluntary, that the information they gave would be kept anonymous, and that their responses to the questions would have no effect on their course grade. Each questionnaire was identified by a 4‐digit number to maintain confidentiality and keep the questionnaire comparable in the pretest and posttest.
3. *Intervention*
  The educational program was conducted over a series of five sessions, each lasting 1.5 h (7.5 h in total) as follows:
  Session I: learning objectives, history, concept and principles of SDH were presented.
  Session II: Models and frameworks related to SDH were presented.
  Session III: Structural determinants of SHD were presented.
  Session IV: Intermediary determinants of SDH were presented.
  Session V: An SDH video clip was shown and further discussed with student participation.
4. *Posttest*

In the posttest step, students' knowledge and attitudes were collected again 1 month after the educational sessions, using the aforementioned questionnaire.

### 2.4 Data Analysis

The Statistical Package for the Social Sciences (SPSS) for Windows, version 25, was used to compute descriptive and inferential statistics (SPSS, Chicago, IL). We first used a nonparametric one‐sample Kolmogorov–Smirnov (KS) test to evaluate the consistency of data scattering in the case of knowledge and attitude scores. Due to the assumptions for the paired *t*‐test was not satisfied [24] (the data scattering patterns were not normal, KS *p*‐value < 0.05), the nonparametric equivalent for paired samples *t*‐test (Wilcoxon Signed‐Ranks Test) was used to compare the knowledge and attitude of students before and after educational intervention. A *p* < 0.05 (two‐sided) was regarded as statistically significant.

#### 2.4.1 Ethics Issues

This study was approved by the Ethics Review Board at the National Agency for Strategic Research in Medical Education (NASR), Tehran Iran (Ethical code number: 960326. To ensure ethical research practices, participants provided written informed consent. This consent form explicitly confirmed the anonymity of participant identities and responses. Additionally, it assured participants that their data would be handled with the utmost confidentiality. All authors have read and approved the final version of the manuscript. The corresponding author had full access to all of the data in this study and takes complete responsibility for the integrity of the data and the accuracy of the data analysis

## 3 Results

This study encompassed 200 students—66 males and 134 females—pursuing various degrees: BSc (64%), MSc (18.50%), MD (10.50%), and PhD (7%). Their ages varied from 18 to 51 years, with an average age of 22.75 years (standard deviation [SD] = 5.05). A majority of the students (85.50%) were single, and nearly half (48.50%) resided in dormitories (Table 1).

**Table 1:** Demographic features of the students, (*n* = 200).

Features		Mean (SD)/frequency (%)
Age		22.75 (5.05)
Sex	Male	66 (33%)
	Female	134 (67%)
Marital status	Single	171 (85.50%)
	Married	29 (14.50%)
Academic degree	BSc	128 (64%)
	MSc	37 (18.50%)
	MD	21 (10.50%)
	PhD	14 (7%)

**Figure 1:**
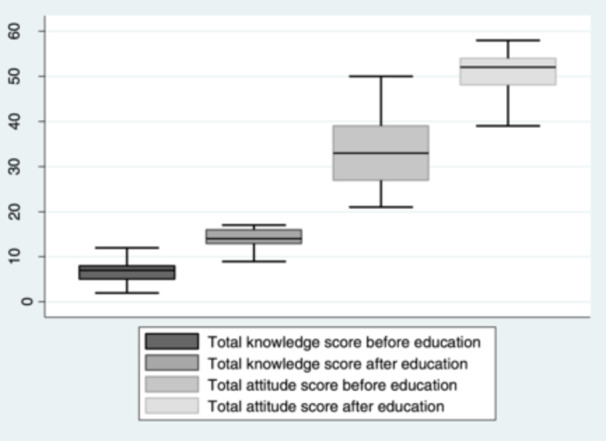
Total knowledge scores and total attitude scores of medical sciences students about social determinants of health (SDH) before and after educational intervention.

Before the educational intervention, the students' total knowledge scores ranged from 2 to 15, and attitude scores from 21 to 50. One month after the pretest and educational intervention, these scores improved significantly, with knowledge scores rising to 4–17 and attitude scores to 20–58. Initially, the average knowledge score was 6.85 (standard deviation [SD] = 2.22). This score increased to 13.83 (SD = 2.67) following the educational intervention. Similarly, the mean attitude score saw a notable increase from 33.64 (SD = 6.89) to 50.09 (SD = 5.36) after a 1‐month interval between the intervention and the posttest evaluation. These results are visually represented in Figure 1.

The statistical analysis using the Wilcoxon Signed‐Ranks Test indicated that the educational intervention on SDH had a significant effect on the students' knowledge and attitudes. The median posttest knowledge scores were notably higher, with a median of 14, compared to the pretest median of 7 (*Z* = −11.89, *p* < 0.001). Similarly, the median posttest attitude scores showed a substantial increase to a median of 52, from the pretest median of 33 (*Z* = −11.60, *p* < 0.001). These findings demonstrate that the educational program led to a statistically significant enhancement in students' knowledge and attitudes regarding SDH, from the baseline to 1‐month postintervention, as detailed in Table 2.

**Table 2:** Comparison of test results between baseline and 1‐month after SDH‐related educational intervention.

	*N* (total)	Median score		*Z*	*p*‐Value
		Baseline	1 month after		
Knowledge	200	7	14	−11.89	<0.001
Attitude	200	33	52	−11.60	<0.001

Abbreviation: SDH, social determinants of health.

## 4 Discussion

The study aimed to investigate the effects of an educational program on medical students' knowledge and attitudes regarding SDH. The findings revealed that there was a significant improvement in both knowledge and attitudes towards SDH 1 month after the educational intervention, compared to the baseline measurements taken before the intervention. This suggests that the educational program was effective in enhancing students' knowledge and attitude on SDH.

University students from diverse backgrounds can attain social accountability by thoroughly grasping topics related to SDH and engaging in pertinent interventions to mitigate negative factors affecting the community's health. The vision of the SDH educational framework can be actualized through the cultivation of relationships between university graduates and community members [25]. This vision can be further enhanced within a learning community characterized by a sense of belonging, mutual understanding, and trust [26].

SDH is crucial for human well‐being, and overlooking them can hinder the attainment of health objectives and the establishment of health equity [27]. In line with the health transformation plan [28], Iran's Ministry of Health, Treatment, and Medical Education has increasingly focused on social factors affecting health to ensure optimal health outcomes for the population. Consequently, a key responsibility of the ministry is to equip medical science students with the knowledge and skills to address societal needs. Education on SDH is fundamental in enhancing the social accountability of students [29, 30].

“The drive to incorporate public health concepts into medical education is steadily gaining momentum, both in academic discourse and practical implementation. In a groundbreaking initiative, Rosenberg et al. [31] conducted an experimental course at Columbia University specifically tailored for medical students. The course aimed to nurture physicians who grasp the wider public health ramifications of clinical interventions, extending their gaze beyond individual patient care [31]. This approach has demonstrated significant benefits by equipping future doctors with the capacity to adeptly identify and tackle public health challenges.”

Kasper et al. [32] meticulously outlined their teaching approach for the course titled “Introduction to Social Medicine and Global Health,” which they delivered to first‐year students at Harvard Medical School. The course was thoughtfully structured into three key sections: foundational concepts in social medicine, exploration of the social implications of disease, and practical applications through translational social medicine. Their emphasis on comprehensiveness underscores how this course equips medical students with essential knowledge, empowering them to adeptly navigate and address complex health issues. Gonzalez et al. [33] conducted a study involving health education sessions for first‐year medical students. The study included 13 sessions, with 39 students enrolled in the Health Disparities and Advocacy course. Both pre‐ and posttests were administered to these students. The curriculum was organized into three main sections: background information, provider contributions to health inequalities and systemic contributions to health disparities (referred to as SDH). The study also used three instructional models including lecture and multimedia presentations, reflective conversations, and skill‐building seminars. Learners showed statistically significant improvements in knowledge, attitudes, and self‐confidence due to the course implementation. Previous research, in alignment with the current study, has shown that using lectures as a teaching method effectively conveys SDH concepts and positively impacts students' knowledge and attitudes. However, it's advisable to complement lectures with hands‐on, practical approaches. In other words, while lectures are valuable, a mix of interactive and experimental methods can enhance the learning experience [34].

Indeed, the application of these methods in both community and clinical settings is instrumental, particularly for students who engage with patients, families, and communities. Teaching SDH in health professions should initially focus on imparting SDH concepts to bolster knowledge. However, as SDH educational programs progress, they should be tailored and implemented according to community needs, enabling students to effectively utilize their acquired knowledge in practical, real‐world situations. This approach ensures that education in SDH is not only theoretical but also pragmatically relevant to the societal contexts' students will serve.

O'Brien et al.'s study [35], which involved 12 students from various disciplines including medicine (nine), public health (one), psychology (one), and Hispanic studies (one), suggested that collaborations between academic medical centers and community‐based organizations could establish a practical, impactful, and enduring framework for educating medical students about social determinants of health. This partnership model is proposed as a means to enhance the training of medical students in understanding and addressing the complex social factors that influence health outcomes. They also demonstrated that students’ learning through this participatory program is more than traditional education programs, and it has created a need to assist underprivileged populations. They suggested that, in addition to satisfying program educational aims, this engagement would promote community health and develop a reputation for community medical academic institutions. DeHaven et al. [36] reported on the development of the Community Health Fellowship Program, aimed at preparing medical students. The findings showed that participants held optimistic attitudes toward the program. Consequently, to build upon the current study, it is recommended that future research should focus on community‐based educational interventions. Such studies could employ experimental methods to facilitate the practical application of knowledge gained by students, thereby reinforcing the link between education and community health improvement.

The study's results underscore the beneficial impact of educational interventions on improving medical students' knowledge and attitudes regarding SDH. This positive effect emphasizes the importance of deliberate educational strategies. Policymakers in medical science education should prioritize the integration of health‐related social concepts into the curriculum. By doing so, we can better equip future healthcare professionals to grasp the broader context of health and wellness. As practical step establishing workshops specifically aimed at assisting curriculum designers and planners is crucial. These workshops can foster creativity and innovation in curriculum development. Ultimately, we must rigorously assess the effectiveness of these curricula and ensuring alignment with educational objectives guarantees that learners benefit optimally. In summary, let us persistently enhance medical education by weaving social awareness throughout the educational process. Our research findings enrich the existing knowledge base in the domain of medical science education in Iran. They emphasize the imperative for revising curricula in health sciences to align with societal requirements and underscore universities' responsibility in advancing public health. Furthermore, our study affirms that educational approaches have the potential to positively influence attitudes and cultivate a constructive mindset among medical students. These pedagogical efforts seek to transition from a narrow, treatment‐focused outlook to a more comprehensive, community‐centered approach to health.

### 4.1 Limitation of Study

We acknowledge that our sample included students from various degree levels: BSc, MSc, PhD, and MD, which may introduce potential bias. However, our deliberate choice aimed to achieve several critical objectives:

1. Holistic Perspective:

- By involving learners across different academic stages, we sought to capture a comprehensive view of medical education.
- Understanding the entire educational continuum—from undergraduate to doctoral levels—enriches our insights.

2. Interdisciplinary Collaboration:

- Real‐world healthcare demands collaboration across disciplines and degrees.
- Our study mirrors this collaborative reality, emphasizing the importance of interdisciplinary training.

3. Generalizability:

- Our findings extend beyond specific degree programs.
- They inform educational strategies applicable to diverse student populations, enhancing the broader field of medical education.

While acknowledging potential bias, we recommend two approaches to mitigate bias in future studies including:

- Conducting subgroup analyses based on degree type to explore differential effects.
- Implementing rigorous statistical controls to address confounding factors related to degree level.

## 5 Conclusion

Educational interventions grounded in SDH have the potential to enhance students' understanding and perspectives. It is essential to embed SDH‐focused educational programs within both medical science curricula and practical training. Such programs should also be reflected in educational policies to ensure accountability, encompass the broader social and nonmedical factors influencing health and disease, reduce health disparities, and tackle the societal issues affecting health status. In Iran's health education policies, there appears to be an incomplete integration of policy and practice guidelines emphasizing SDH. To bridge this gap, an effective strategy might involve organizing consensus‐building meetings among researchers, policymakers, and health sector leaders to establish a widely accepted framework for incorporating SDH into educational content. Subsequently, the SDH concepts mastered by university graduates could be applied to future community health initiatives, thereby promoting healthier outcomes for society at large.

## Author Contributions

**Mostafa Amini‐Rarani:** conceptualization, methodology. **Athar Omid:** writing–review and editing. **Mehdi Nosratabadi:** writing–review & editing, conceptualization, methodology.

## Conflicts of Interest

The authors declare no conflicts of interest.

## Transparency Statement

The lead author Mehdi Nosratabadi affirms that this manuscript is an honest, accurate, and transparent account of the study being reported; that no important aspects of the study have been omitted; and that any discrepancies from the study as planned (and, if relevant, registered) have been explained.

## Data Availability

The data that support the findings of this study are available on request from the corresponding author. The data are not publicly available due to privacy or ethical restrictions. Data are available on reasonable request. The data that support the findings of this study are available from the corresponding author.
